# Oral Microbiota Profile of Individuals Who Abuse Methamphetamine

**DOI:** 10.3389/fcimb.2021.706961

**Published:** 2021-09-10

**Authors:** Yongde Yang, Xuan Yu, Xue Yang, Kuan Zeng, Guangya Liu, Wei Hao, Sheng Zhang, Gang Wang

**Affiliations:** ^1^Wuhan Mental Health Center, The Ninth Clinical School, Tongji Medical College, Huazhong University of Science and Technology, Wuhan, China; ^2^Department of Pathophysiology, School of Basic Medicine, Key Laboratory of Education Ministry of China for Neurological Disorders, Tongji Medical College, Huazhong University of Science and Technology, Wuhan, China; ^3^Wuhan Jin-yintan Hospital, Wuhan, China; ^4^Department of Psychiatry, The Second Xiangya Hospital, Central South University, Changsha, China

**Keywords:** 16S rRNA sequencing, oral microbiota, methamphetamine, addiction, oral health

## Abstract

The poor oral health condition of individuals who abuse methamphetamine (MA) is well known. The roles of the oral and fecal microbiomes in addiction and nervous system diseases have been the focus of many studies. However, changes in the microbiota composition of MA users have not been reported. This was addressed in the present study in 20 MA users and 14 sex-matched healthy subjects. Saliva samples were collected and high-throughput 16S rRNA sequencing and bioinformatic analysis were performed to evaluate oral microbiome profiles. The results showed that species richness was significantly lower in the MA group than in the control group. Bacterial taxa that are known to be related to oral diseases such as Negativicutes, Veillonellaceae, Veillonella, and Selenomonadales had higher relative abundance in the MA group than in the control group, and the relative abundance of Prevotella melaninogenica—a putative etiologic agent of periodontal disease—was also higher. Avoiding MA use and improving oral hygiene practices over a short term (i.e., during hospitalization for 2 weeks) did not alter the oral microbiota composition of MA users. Although the causal relationship between changes in oral microbiome profile and MA abuse remains to be determined, our results suggest that oral disease prevention and treatment strategies are important for MA users.

## 1 Introduction

Oral health problems are common in injection drug users but there is a lack of a specific treatments (Laslett et al., 2008). Individuals who abuse methamphetamine (MA)—a highly addictive drug—frequently have xerostomia, caries, poor oral hygiene, and excessive tooth wear as well as low oral health-related quality of life (Donaldson and Goodchild, 2006; Hamamoto and Rhodus, 2009; Mukherjee et al., 2018). However, the relationship between oral health problems and MA addiction or MA-induced psychosis is not well understood.

The oral microbial ecosystem plays an essential role in maintaining oral health; dysbiosis of the oral microbiome has been shown to be involved in various oral diseases and can induce periodontitis (Minty et al., 2019). There is also increasing evidence that it is related to systemic diseases and mental health disorders (Maitre et al., 2020). Periodontal bacteria have been linked to Alzheimer disease and dementia (Riviere et al., 2002; Rai et al., 2012; Sochocka et al., 2017); among them, Porphyromonas gingivalis and/or its product gingipain have been detected in the brain (Poole et al., 2013; Ilievski et al., 2018). In addition, patients with bipolar disorder have a higher total bacterial load (Cunha et al., 2019), and dysbiosis of the oral microbiome has been reported in patients with neurologic and developmental disorders, including autism spectrum disorder (Hicks et al., 2018; Qiao et al., 2018; Kong et al., 2019). Thus, oral microbiota are potential targets for the prevention and treatment of oral as well as systemic and neurologic diseases (Zhang et al., 2018).

Oral bacteria can alter fecal microbiota composition by colonizing the intestine. Several studies have reported differences in gut microbiome profile between MA users or MA-treated animals and healthy controls (Ning et al., 2017; Cook et al., 2019). However, there have been no studies comparing the oral microbiota composition of MA users and healthy subjects. To this end, in this study we investigated changes in the oral microbiome of MA-addicted individuals by high-throughput 16S rRNA sequencing and bioinformatic analysis. We also analyzed the oral microbiome profile of this group after hospitalization in order to determine whether it would be affected by drug withdrawal.

## 2 Materials and Methods

### 2.1 Subject Selection

The Ethics Committee of Wuhan Mental Health Center (Wuhan, Hubei, China) approved the study protocol. All participants provided written, informed consent prior to enrollment after receiving a written description of the study.

We recruited 20 subjects with a clinical diagnosis of MA addiction (mean age, 35 ± 8.09 years; range, 20–55 years) along with 14 sex-matched healthy control subjects who had never used MA or any other addictive drug (mean age, 29 ± 4.97 years; range, 22–39 years). The group of MA addictive individuals is named as the MA group, and the group of control subjects is named Con groups. All of the subjects were male; there were no differences between groups in terms of marital status and education level; and all of the subjects belonged to the same ethnic group (Han Chinese), lived in Wuhan, and had similar diets. None of the subjects had taken antibiotics, probiotics, or prebiotics in the 3 months prior to sample collection, and none were taking anti-inflammatory or antioxidant drugs. None of the subjects abused alcohol.

Information on MA use was collected from subjects in the MA group. The mean age of initial MA use was 26.8 ± 7.06 years (range, 12–40 years); mean duration of MA use was 8.5 ± 2.80 years (range, 3–15 years); and mean frequency of MA use was 4.3 ± 1.38 days per week. The subjects in the MA group had been hospitalized 1–6 times for their addiction (**Table 1**).

**Table 1:** Characteristics of the study population.

		MA	Con
Subjects (n)		20	14
Proportion of Males, No. (%)		16 (100%)	14 (100%)
Age (years; means ± SD) (range)		35 (8.09) (20-55)	29(4.97) (22-39)
Employed, No. (%)		3 (15%)	11 (78.57%)
Education	primary school, No. (%)	1(5.00%)	1 (7.14%)
	middle school, No. (%)	7 (35.00%)	6 (42.86%)
	high school, No. (%)	8 (40.00%)	4 (28.57%)
	university or higher, No. (%)	4 (20.00%)	3(21.43%)
Married, No. (%)		12 (60.00%)	9 (64.29%)
Age of initial METH use (years; means ± SD) (range)		26.8 (7.06) (12-40)	NA
Duration of METH use (years; means ± SD) (range)		8.5(2.80) (3-15)	NA
Frequency of METH use (days per week; means ± SD) (range)		4.3 (1.38) (3-7)	NA
Times of hospitalization (times; means ± SD) (range)		2 (1.77) (1-6)	NA

All of the subjects (in both MA and Con groups) smoked cigarettes daily but did not abuse any other addictive substances. None of the subjects had a preference for high-sugar beverages or food. We collected saliva samples from the MA group at the time of admission and after they had been hospitalized for 2 weeks. The group of samples from MA group after their hospitalized treatment were called AH group. During this period, they had no access to MAs or alcohol and were provided the same food at regular intervals (3 times a day). They were also treated with olanzapine (10–20 mg/day) according to the doctor’s advice, and brushed their teeth twice a day (in the morning before eating breakfast and in the evening before sleeping).

### 2.2 Sample Collection and DNA Isolation

Saliva samples were collected from both groups in the early morning before any oral hygiene practice. The subjects were asked to refrain from eating or drinking for at least 3 h prior to sample collection. Saliva (1–3 ml) was collected in sterile DNA- and RNA-free Eppendorf tubes and stored at −80°C until use. DNA was extracted from the samples using the Magnetic Soil and Stool DNA Kit (Tiangen, Beijing, China) according to the manufacturer’s instructions. DNA purity and concentration were determined by agarose gel eletrophoresis. DNA samples were diluted to 1 ng/μl with sterile water.

### 2.3 PCR of 16S rRNA Gene

Specific primers with barcodes (16S V4: 515F and 806R) were used for PCR amplification of the bacterial 16S rRNA gene. The 30-μl reaction contained 15 μl Phusion High-Fidelity PCR Master Mix (New England Biolabs, Ipswich, MA, USA), 0.2 μM forward and reverse primers, and ~10 ng of DNA template. The thermal cycling program was run on a T100 gradient PCR instrument (Bio-Rad, Hercules, CA, USA) and consisted of the following steps: initial denaturation at 98°C for 1 min; 30 cycles of denaturation at 98°C for 10 s, annealing at 50°C for 30 s, and elongation at 72°C for 30 s; and final extension at 72°C for 5 min.

The PCR products were mixed with the same volume of SYBR Green 1× loading buffer and visualized by electrophoresis on a 2% agarose gel. Bands between 400–450 bp were excised and mixed in equidensity ratios and purified with a GeneJET Gel Extraction Kit (Thermo Fisher Scientific, Waltham, MA, USA).

### 2.4 Library Preparation and Sequencing

Sequencing libraries were generated with a TruSeq DNA PCR-Free Library Preparation Kit (Illumina, San Diego, CA, USA) and index codes were added. Library quality was assessed with a Qubit@ 2.0 fluorometer (Thermo Fisher Scientific) and Bioanalyzer 2100 system (Agilent, Santa Clara, CA, USA). The library was sequenced on an Illumina NovaSeq platform as 250-bp paired-end reads.

### 2.5 Data Analysis

Paired-end reads from the original DNA fragments were merged with FLASH (https://ccb.jhu.edu/software/FLASH/W) and assigned to each sample according to the unique barcodes. Sequences were analyzed with the Quantitative Insights Into Microbial Ecology (QIIME) v1.7.0 software package and in-house Perl scripts were used to analyze alpha (within samples) and beta (among samples) diversity. Reads were first filtered using QIIME quality filters; an OTU table was then generated with the pick_de_novo_otus.py workflow script to identify operational taxonomic units (OTUs) comprising sequences with ≥97% similarity. Representative sequences of each OTU were selected and annotated with the Ribosomal Database Project classifier. All the analyses below were based on the OUT results. Alpha diversity indices were calculated with R v2.15.3 software (R Foundation for Statistical Computing, Vienna, Austria). The rarefaction curve reflects the rationality of sequencing data volume and the richness of species in samples. Beta diversity was used for comparing the microbiome composition between the two groups. Both weighted and unweighted UniFrac distances, which belong to beta diversity measures, were calculated with QIIME. MetaStat and linear discriminant analysis (LDA) effect size (LEfSe) method were used to estimate differences in effect size of each taxon. Analysis of molecular variance (AMOVA) is a nonparametric analysis, which tests the significance of differences between groups based on weighted and unweighted UniFrac distances (Lutz et al., 1996). MaAsLin (Multivariate Analysis by Linear Models) were used to determine age-associated taxa (Xiuying et al., 2021). MetaStat, LEfSe and AMOVA analyses were carried out with R software, Version 2.15.3. The MaAsLin analysis was processed through the MaAsLin online tool (https://huttenhower.sph.harvard.edu/galaxy/). All significance tests were 2-sided, and P<0.05 was considered significant.

## 3 Results

### 3.1 Oral Bacterial Community Structure Differs Between MA Users and Healthy Subjects

We analyzed the oral microbiota of the MA and Con groups by 16S rRNA gene amplicon sequencing. The ecologic features of oral bacterial communities were evaluated by various indices at the OTU level with a 97% consistency threshold. Good’s coverage and species accumulation boxplots indicated reasonable amounts of sequencing data (**Table 2**, **Supplementary Figure 1**). The rarefaction curve and rank abundance were used to evaluate the diversity of samples within each group (**Supplementary Figure 1**). The rarefaction curve, which is based on observed species, reflected most of the bacterial taxa in the oral cavity, but the species richness in the MA group was significantly lower than that in the Con group (**Table 2**). This was supported by the Chao and abundance-based coverage estimator (ACE) indices (**Table 2**). OTU analysis showed a longer tail in the rank abundance curve of the Con group as compared to the MA group (**Supplementary Figure 1**), indicating that the former had more OTUs present at low abundance, which could partly explain the differences in species richness between the 2 groups. The Shannon and Simpson indices indicated that there was no difference in bacterial community diversity between groups (**Table 2**).

**Table 2:** Comparison of species richness and diversity estimation on OUT levels.

Group	Observed species	Good’s coverage	Richness estimator				Diversity index	
			ACE	95%CI	Chao 1	95%CI	Shannon	Simpson
Con	551	0.997	701.184	603.5-798.9	678.247	587.7-768.8	4.837	0.877
MA	427**	0.998	517.19**	461.8-572.6	501.23**	448.5-553.9	4.672	0.879

**p < 0.01, Wilcoxon rank-sum test.

All the indexes were analyzed under the 97% consistency threshold of OTUs for different samples.

### 3.2 Oral Microbiota Composition Is Altered in MA Users

**Figure 1:**
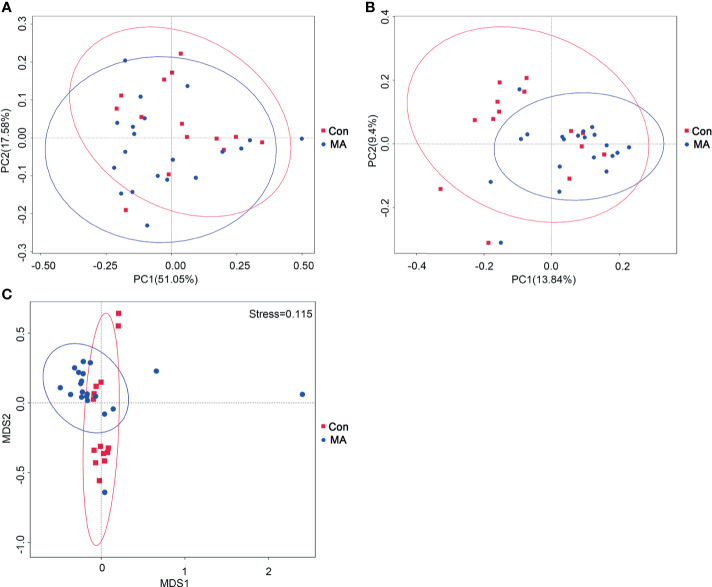
MA abuse is associated an altered oral microbiome. **(A, B)** Principal coordinate analysis (PCoA) of bacterial beta diversity based on unweighted **(A)** and weighted **(B)** UniFrac distances. **(C)** Nonmetric multidimensional scaling plot based on Bray–Curtis distances. MA and Con groups are colored in red and blue, respectively.

To compare microbial community composition between groups, beta diversity was calculated based on weighted and unweighted UniFrac distances. The oral microbiotas in the 2 groups could be divided into clusters according to community composition using weighted and unweighted UniFrac and Bray–Curtis metrics (**Figure 1A, B**). The analysis of molecular variance (AMOVA) statistic based on unweighted UniFrac and multi response permutation procedure statistic based on Bray–Curtis metrics confirmed the significant differences in microbiota community composition between the MA and Con groups (**Figure 1C**, **Supplementary Table 2**); however, the AMOVA statistic based on weighted UniFrac distances showed that there were no significant differences (**Supplementary Table 2**); these may have been masked by the low abundance of oral microbiota communities. To complement the clustering results, bacterial richness was analyzed with a Venn diagram; 1152 OTUs were common to both groups, 479 were detected only in the MA group, and 337 were present only in the Con group (**Supplementary Figure 1**).

### 3.3 Different Bacterial Taxa Are Present in MA Users and Healthy Subjects

**Figure 2:**
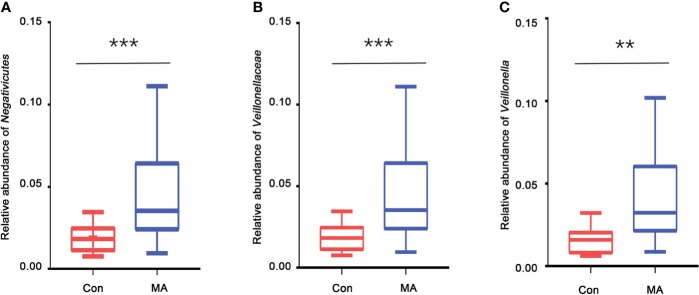
Taxa showing significant differences in relative abundance between MA users and healthy subjects. **(A–C)**. Relative abundance of Negativicutes (class level) **(A)**, Veillonellaceae (family level) **(B)**, and Veillonella (genus level) **(C)** in the MA and Con groups. **p < 0.01, ***p < 0.001, Wilcoxon rank-sum test.

**Figure 3:**
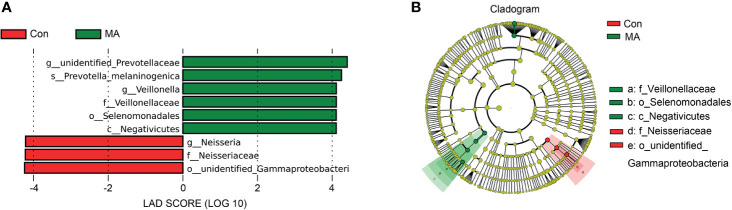
Differences in oral microbiota composition between MA users and healthy subjects. **(A)** Cladograms generated by LEfSe showing taxonomic differences between MA and Con groups. Nodes in red and green represent taxa that are less and more abundant in the MA group relative to the Con group, respectively. **(B)** Linear discriminant analysis (LDA) scores for bacterial taxa differing in abundance between the MA and Con groups. Positive and negative LDA scores indicate taxa enriched in the MA and Con groups, respectively. Only taxa with P < 0.01 (Wilcoxon rank-sum test) and LDA >4.0 are shown.

MetaStats analysis was carried out to investigate differences in oral microbiota composition between the MA and Con groups. No taxa at the phylum or order level differed significantly between the 2 groups. At the class level, the relative abundance of Negativicutes was significantly higher in the MA group than in the Con group (**Figure 2A**, **Supplementary Table 3**). Four families were differentially represented between the 2 groups: the relative proportions of Veillonellaceae and Cryptosporangiaceae were significantly higher in the MA group than in the Con group, with the latter found only in MA users (**Figure 2B**, **Supplementary Table 3**). Meanwhile, undefined Spirochaetes and Thermomonosporaceae were not detected in the MA group. There were 19 genera that differed significantly between groups including 1 that was predominant (i.e., accounting for >1% of total sequences in one of the groups): Veillonella was more abundant in MA users than in healthy subjects (**Figure 2C** and **Supplementary Table 3**). LEfSe analysis was carried out to further analyze the bacterial community structure (**Figure 3A**) and linear discriminant analysis (**Figure 3B**) was used to estimate differences in effect size of each taxon in the 2 groups. The results of the LEfSe analysis showed that the relative abundance of undefined Gammaproteobacteria, Neisseriaceae, and Neisseria was reduced whereas that of Negativicutes, Selenomonadales, Veillonellaceae, Veillonella, undefined Prevotellaceae, and Prevotella melaninogenica was increased in MA users compared to Con subjects (**Figure 3**). However, considering the possible effects of age on the oral bacteria, MaAsLin was performed to determine age-associated taxa. The relative abundance of unidentified Gammaproteobacteria was significantly associated with age (coefficient = 0.0024, q value= 0.0345) (**Supplementary Table 3**).

### 3.4 Short-Term Avoidance of MA Does Not Alter Oral Microbiota Composition

**Figure 4:**
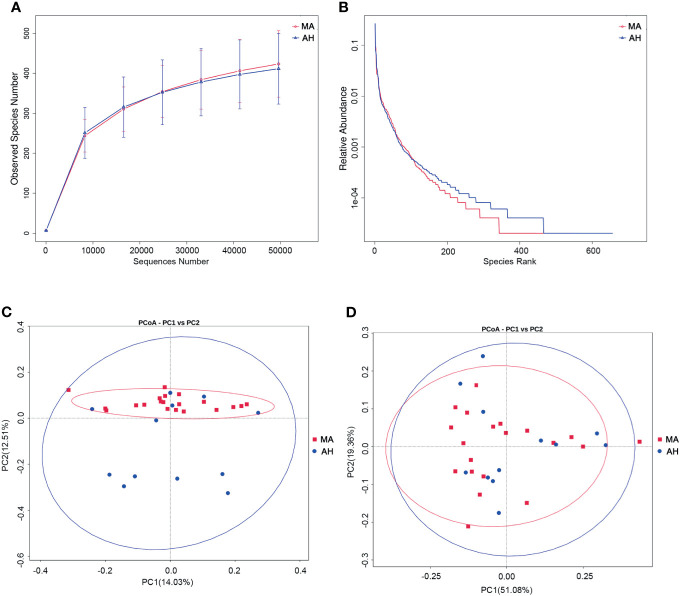
Abstaining from MA for a short term does not restore oral microbiome profile in MA users. **(A, B)** Rarefaction curves **(A)** and rank abundance curves **(B)** of the MA and AH groups. **(C, D)**. Microbial community composition in MA and AH groups evaluated by principal coordinate analysis (PCoA) based on beta diversity, including weighted UniFrac distances **(C)**, and unweighted UniFrac distances **(D)**.

We examined the oral microbiota composition of MA users after they had stopped using MA to determine whether it could be restored by abstinence. However, only 12 of the MA users agreed to participate in this part of the study. We analyzed samples that were collected after the MA users had been at the hospital for 2 weeks. During this period, their diet was consistent and they received treatment with olanzapine (10–20 mg/day). There were no differences in species richness between the MA and AH groups based on the rarefaction curve (**Figure 4A**), which was also confirmed by Chao and ACE indices (**Supplementary Table 4**). The rank abundance curve indicated similar species abundance and evenness between the MA and AH groups, but there were more species with low abundance in the AH group (**Figure 4B**). The Shannon and Simpson indices revealed no difference in community diversity between the 2 groups (**Supplementary Table 4**). The results of the principal coordinate analysis based on beta diversity (weighted and unweighted UniFrac distances) showed that there was no difference in microbial community composition between groups (**Figures 4C, D**), which was confirmed by AMOVA (**Supplementary Table 5**). Further, the results of the MetaStats analysis showed no differences in abundance at the phylum level. However, the relative abundance of several species was altered in the MA group and at the class level, Ignavibacteria and Chloroflexia were more abundant whereas Fibrobacteria was less abundant in MA users. All of these classes were less predominant (<0.01% of total sequences in both groups). At the order, family, and genus levels, taxa showing altered abundance were all less predominant (**Supplementary Table 5**). Taken together, these results demonstrate that changes in oral microbiota induced by MA cannot be reversed in a short period of time even with good oral hygiene practices, and that olanzapine treatment does not influence oral microbiota composition (**Supplementary Table 6**).

## 4 Discussion

We carried out a comparative analysis of oral microbiota in individuals who abuse MA and healthy subjects by 16S rRNA gene sequencing. Bacterial species richness was lower in the MA group than in the Con group, and the latter had more species present at low abundance. Several bacterial taxa that are known to be related to oral diseases had higher relative abundance in the MA group. The composition of oral microbiota composition did not alter after the two-weeks hospitalized treatment of MA users. These results suggest that oral disease prevention and treatment strategies are important for MA users.

Oral microbiota composition is affected by host immune competence and diet. In some disease states or upon exposure to noxious environmental factors, byproducts of metabolism and the immune response alter the oral environment, which can lead to oral dysbiosis (Lamont et al., 2018). This is associated not only with oral diseases such as dental caries and periodontal disease, but also with metabolic (e.g., cardiovascular disease and dyslipidemia) and neurologic (ego, Alzheimer disease) disorders (Kuboniwa et al., 2012; Minty et al., 2019; Sureda et al., 2020). Among individuals who abuse MA, there is a higher prevalence of untreated dental caries, periodontitis, and severe periodontitis as compared to healthy subjects (Hegazi et al., 2021), which is associated with an altered microbiome profile. Chronic MA use and poor oral hygiene can compromise physiologic barriers, resulting in infections (Graves et al., 2019).

P. gingivalis plays an important role in chronic periodontitis and is detected in the brain of patients with Alzheimer disease (Dominy et al., 2019). Therapeutic targeting of this bacterium was shown to reduce neuroinflammation and rescued neurons in the hippocampus, suggesting a link between oral microbiota and neuronal damage that can serve as a basis for treatment. An open question is whether there is a relationship between gut and oral microbiomes in MA users.

The lower bacterial species richness in the MA group seemed similar with the situation of fecal microbiota. We’ve found that MA users have lower diversity of fecal microbiota than healthy individuals (manuscript submitted for publication), which is in line with the results of the present work as microbial ecosystems in different parts of the body may interact. Although the lower species richness in MA users was at odds with the increased diversity of the microbiome in periodontal diseases, the relative abundance of Negativicutes and Veillonellaceae was higher in this group, which was shown to be associated with periodontitis (Griffen et al., 2012; Lamont et al., 2018). Cryptosporangiaceae, which belongs to the Frankiales order and is present in the environment (Wang et al., 2020), was only detected in MA users and may be related to their poor oral hygiene practices. In addition, the relative abundance of Veillonella and Selenomonadales—which are enriched in caries-affected plaques—was higher in the MA group than in the Con group. The same was true for P. melaninogenica, which is not only a putative etiologic agent of periodontal disease but is associated with the occurrence of Alzheimer disease (Allison and Hillman, 1997; Beydoun et al., 2020).

The consumption of sugary beverages and poor oral hygiene are the major reasons for oral diseases in MA users (Clague et al., 2017). We therefore investigated whether the oral microbiome profile of the MA group would change after abstinence from MA and improvement of oral hygiene practices. Following hospital admission, individuals in the MA group were denied access to MA, brushed their teeth twice daily, and ate 3 healthy regular meals a day. They were also treated with olanzapine according to the doctor’s advice. However, after 2 weeks, there were no changes in the diversity and richness of their oral microbiota; only some less predominant classes of bacteria were altered and these are not known to be associated with human diseases. We also found no evidence that olanzapine affected the oral health or oral microbiome of the MA group. It is possible that differences would be observed if the treatment were to be continued over a longer period of time.

Our results showed that bacterial taxa with a higher relative abundance in the MA group were mostly related to periodontal diseases and dental caries, suggesting that the higher incidence of oral diseases in MA users is associated with alterations in pathogenic bacteria. However, it is difficult to establish the cause/effect relationship between changes in oral microbiota and oral disease given the poor hygiene practices and medical condition of MA users. The higher relative abundance of P. melaninogenica in the MA group may also indicate a link between oral microbiota and cognitive function, although there has been little research on this topic. The fact that oral microbiota composition in MA users was unchanged after hospital admission highlights the importance of oral disease prevention and treatment in these individuals. Finally, the question of whether poor oral health aggravates oral dysbiosis and MA-induced disorders such as cognitive dysfunction warrants further study.

There were also several limitations of this research, including the small number of samples in each group and the single-gender of the individuals recruited in the research. It mainly limited by the situation that the number of patients we could contact with is not very large and they were mainly males. The further research would expand and find the differences of oral microbiome between female MA abusers and control subjects.

## Data Availability Statement

The datasets presented in this study can be found in online repositories. The names of the repository/repositories and accession number(s) can be found below: Bioproject SRA, PRJNA728576.

## Ethics Statement

The studies involving human participants were reviewed and approved by The Ethics Committee of Wuhan Mental Health Center (Wuhan, Hubei, China). The patients/participants provided their written informed consent to participate in this study.

## Author Contributions

YY collected the samples and clinical data. XYu performed the data analysis and original draft writing. XYa, GL, and KZ searched for the enrolled subjects and performed data curation. WH revised the draft. SZ and GW came up with conception and design of the study. All authors contributed to the article and approved the submitted version.

## Funding

The study was supported by the National Key Research and Development Program of China (2018YFC1314303), Youth Program of Hubei Province Nature Science Foundation (2018CFB334), Wuhan medical research program (WX19Y22, WX19Z31, and WX18Q41).

## Conflict of Interest

The authors declare that the research was conducted in the absence of any commercial or financial relationships that could be construed as a potential conflict of interest.

## Publisher’s Note

All claims expressed in this article are solely those of the authors and do not necessarily represent those of their affiliated organizations, or those of the publisher, the editors and the reviewers. Any product that may be evaluated in this article, or claim that may be made by its manufacturer, is not guaranteed or endorsed by the publisher.
