# Supplementary material for: Oral Microbiota Profile of Individuals Who Abuse Methamphetamine
Source: Front Cell Infect Microbiol. 2021 Sep 10;11:706961. doi: 10.3389/fcimb.2021.706961 (PMC8461105; doi:10.3389/fcimb.2021.706961)
Supplement: Supplementary file 1 [file Table_1.docx]

**
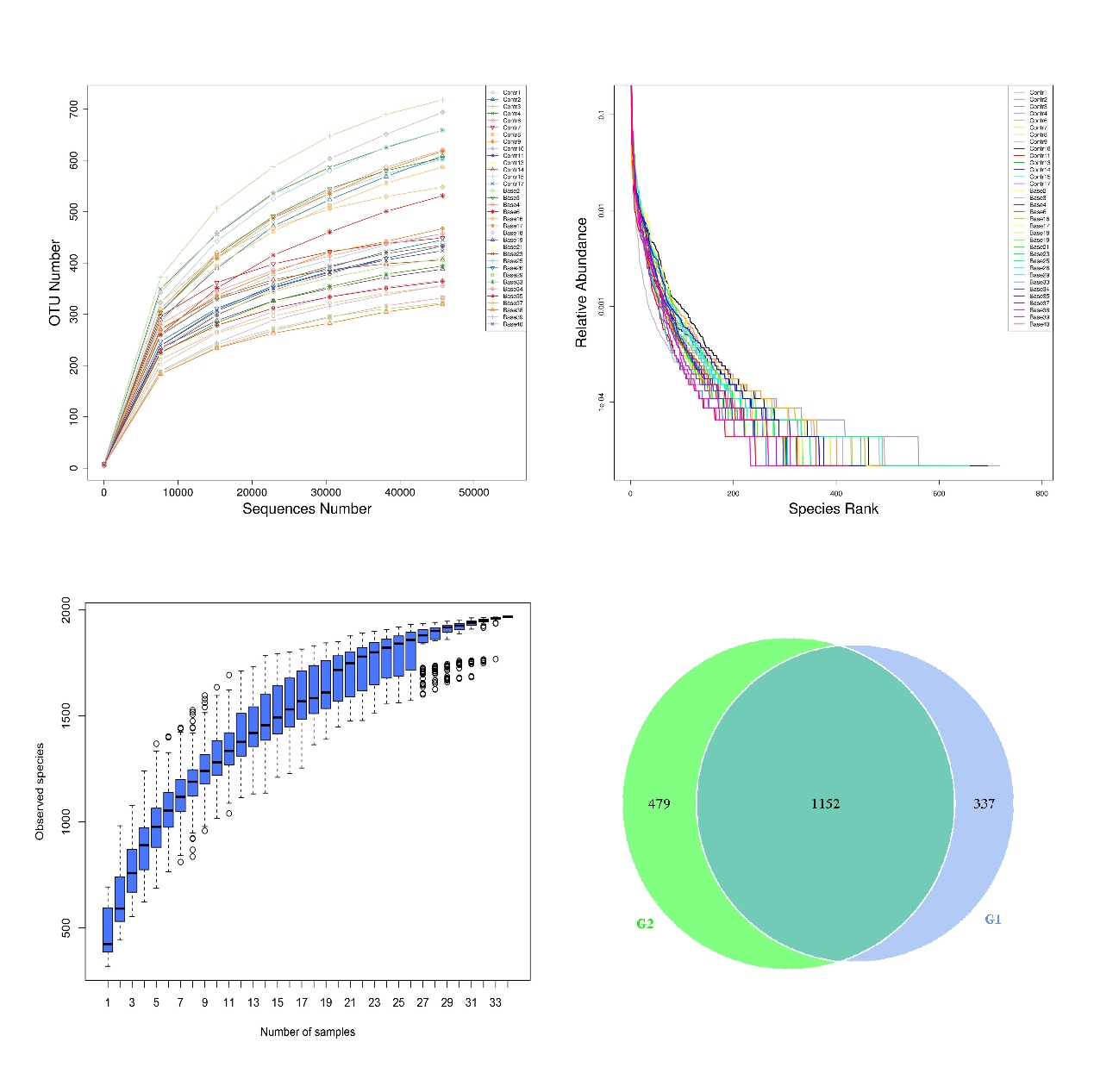
**

**Supplementary Fig 1 Overall structure of the oral bacterial communities.**

**(A)** The rarefaction curve of the number of OTUs. (B**)** Rank abundance of the MA and Con groups. (**C)** The species accumulation boxplot of the MA and Con groups. **(D)**Venn diagram based on OTU clustering. G2 represents Con group, as well as G1 represents MA group.

**Supplementary Table 1** The results of AMOVA statistic based on unweighted/weighted UniFrac and MRPP statistic based on Bray-Curtis metrics.

| unweighted_unifrac_amova | | | | | |
| --- | --- | --- | --- | --- | --- |
| vs_group | SS | df | MS | Fs | p-value |
| MA-Con | 0.291589(4.65058) | 1(32) | 0.291589(0.145331) | 2.00638 | 0.001* |
|  |  |  |  |  |  |
| weighted_unifrac_amova | | | | | |
| vs_group | SS | df | MS | Fs | p-value |
| MA-Con | 0.0881679(1.84399) | 1(32) | 0.0881679(0.0576247) | 1.53004 | 0.197 |
|  |  |  |  |  |  |
| MRPP | | | | |  |
| Group | A | observed-delta | expected-delta | Significance |  |
| MA-Con | 0.0187 | 0.4077 | 0.4155 | 0.042 |  |

**Supplementary Table 2** MetaStat of microbes with significant difference between the MA and Con groups.

| class level |  |  |  |  |  |  |  |  |
| --- | --- | --- | --- | --- | --- | --- | --- | --- |
| Taxa | mean(MA) | variance(MA) | standard error(MA) | mean(Con) | variance(Con) | standard error(Con) | p value | q value |
| c__Negativicutes; | 0.019033357 | 6.54E-05 | 0.002161944 | 0.0442013 | 0.000769373 | 0.006202309 | 0.000999 | 0.043956 |
|  |  |  |  |  |  |  |  |  |
|  |  |  |  |  |  |  |  |  |
| famliy level |  |  |  |  |  |  |  |  |
| Taxa | mean(MA) | variance(MA) | standard error(MA) | mean(Con) | variance(Con) | standard error(Con) | p value | q value |
| f__Veillonellaceae; | 0.018914682 | 6.65E-05 | 0.002179131 | 0.04418818 | 0.000769052 | 0.006201015 | 0.000999 | 0.046703 |
| f__unidentified_Spirochaetes; | 1.87E-05 | 4.92E-09 | 1.87E-05 | 0 | 0 | 0 | 2.38E-05 | 0.004442 |
| f__Cryptosporangiaceae; | 0 | 0 | 0 | 1.64E-05 | 2.86E-09 | 1.20E-05 | 0.000387 | 0.024094 |
| f__Thermomonosporaceae; | 1.41E-05 | 4.86E-10 | 5.89E-06 | 0 | 0 | 0 | 0.00034 | 0.024094 |

| genus level |  |  |  |  |  |  |  |  |
| --- | --- | --- | --- | --- | --- | --- | --- | --- |
| Taxa | mean(MA) | variance(MA) | standard error(MA) | mean(Con) | variance(Con) | standard error(Con) | p value | q value |
| g__Veillonella; | 0.015502762 | 5.69E-05 | 0.002016179 | 0.04060621 | 0.000702431 | 0.005926343 | 0.001998 | 0.044258 |
| g__Mangrovibacter; | 0.00029825 | 3.99E-08 | 5.34E-05 | 8.96E-05 | 5.93E-09 | 1.72E-05 | 0.000999 | 0.032199 |
| g__Sediminispirochaeta; | 1.56E-06 | 3.41E-11 | 1.56E-06 | 4.92E-05 | 7.94E-09 | 1.99E-05 | 0.000999 | 0.032199 |
| g__unidentified_Spirochaetes; | 1.87E-05 | 4.92E-09 | 1.87E-05 | 0 | 0 | 0 | 2.38E-05 | 0.009954 |
| g__Fodinicola; | 0 | 0 | 0 | 1.64E-05 | 2.86E-09 | 1.20E-05 | 0.000387 | 0.0309 |
| g__Ruminiclostridium; | 1.56E-05 | 3.41E-09 | 1.56E-05 | 1.09E-06 | 2.39E-11 | 1.09E-06 | 0.000964 | 0.032199 |
| g__Ferruginibacter; | 1.56E-06 | 3.41E-11 | 1.56E-06 | 2.08E-05 | 3.39E-09 | 1.30E-05 | 0.000442 | 0.0309 |
| g__Syntrophococcus; | 1.72E-05 | 2.81E-09 | 1.42E-05 | 0 | 0 | 0 | 5.77E-05 | 0.012087 |
| g__Slackia; | 2.03E-05 | 2.17E-09 | 1.24E-05 | 3.28E-06 | 6.41E-11 | 1.79E-06 | 0.001504 | 0.037069 |
| g__unidentified_Nitrosomonadaceae; | 0 | 0 | 0 | 1.64E-05 | 2.26E-09 | 1.06E-05 | 0.000387 | 0.0309 |
| g__Thermomonas; | 0 | 0 | 0 | 1.42E-05 | 1.93E-09 | 9.81E-06 | 0.001201 | 0.035945 |
| g__Candidatus_Soleaferrea; | 2.19E-05 | 1.84E-09 | 1.15E-05 | 0 | 0 | 0 | 0.000999 | 0.032199 |
| g__Glutamicibacter; | 1.25E-05 | 9.35E-10 | 8.17E-06 | 0 | 0 | 0 | 0.000826 | 0.032199 |
| g__Dactylosporangium; | 2.03E-05 | 1.14E-09 | 9.01E-06 | 3.28E-06 | 2.15E-10 | 3.28E-06 | 0.001504 | 0.037069 |
| g__Luteimonas; | 2.03E-05 | 1.14E-09 | 9.01E-06 | 3.28E-06 | 2.15E-10 | 3.28E-06 | 0.001504 | 0.037069 |
| g__Serratia; | 1.25E-05 | 6.41E-10 | 6.77E-06 | 0 | 0 | 0 | 0.000826 | 0.032199 |
| g__unidentified_Pyrinomonadaceae; | 1.25E-05 | 7.88E-10 | 7.50E-06 | 0 | 0 | 0 | 0.000826 | 0.032199 |
| g__Bowmanella; | 1.09E-05 | 5.70E-10 | 6.38E-06 | 0 | 0 | 0 | 0.002007 | 0.044258 |
| g__unidentified_Alphaproteobacteria; | 1.87E-05 | 5.04E-10 | 6.00E-06 | 1.09E-06 | 2.39E-11 | 1.09E-06 | 0.000191 | 0.026738 |

**Supplementary Table 3 Multivariate analysis of age and significant different bacteria by linear models.**

| Variable | Feature | Coefficient | N | N not 0 | *p* value | *q* value |
| --- | --- | --- | --- | --- | --- | --- |
| Age | unidentified_Gammaproteobacteria | 0.002437 | 34 | 34 | 0.012077 | 0.034506 |
| Age | Neisseriaceae | 0.002364 | 34 | 34 | 0.020758 | 0.051894 |
| Age | Veillonellaceae | 0.002308 | 34 | 34 | 0.139593 | 0.206987 |
| Age | Negativicutes | 0.002276 | 34 | 34 | 0.144891 | 0.206987 |
| Age | Selenomonadales | 0.002276 | 34 | 34 | 0.144891 | 0.206987 |
| Age | Veillonella | 0.002191 | 34 | 34 | 0.162685 | 0.216913 |
| Age | unidentified_Prevotellaceae | 0.003127 | 34 | 34 | 0.299198 | 0.373998 |
| Age | Neisseria | 0.001894 | 34 | 34 | 0.408652 | 0.480767 |
| Age | Prevotella_melaninogenica | 0.001561 | 34 | 34 | 0.562625 | 0.625139 |
| Age | Cryptosporangiaceae | 1.45E-05 | 34 | 2 | 0.859669 | 0.904915 |

**Supplementary Table 4 Comparison of species richness and diversity estimation on OUT levels of MA and AH groups.**

| Group | observed species | shannon | simpson | chao1 | ACE | goods coverage |
| --- | --- | --- | --- | --- | --- | --- |
| MA | 423 | 4.644 | 0.879 | 481.788 | 497.336 | 0.998 |
| AH | 411 | 4.487 | 0.871 | 453.742 | 464.691 | 0.998 |

All the indexes were analyzed under the 97% consistency threshold of OTUs for different samples.

**Supplementary Table 5** The results of AMOVA statistic based on weighted UniFrac of MA and AH groups.

| vs_group | SS | df | MS | Fs | p-value |
| --- | --- | --- | --- | --- | --- |
| MA-AH | 0.0243224(1.40596) | 1(30) | 0.0243224(0.0468653) | 0.518986 | 0.69 |

| Class level |  |  |  |  |  |  |  |  |
| --- | --- | --- | --- | --- | --- | --- | --- | --- |
| Taxa | mean(MA) | variance(MA) | standard error(MA) | mean(AH) | variance(AH) | standard error(AH) | p value | q value |
| c__Ignavibacteria; | 1.71E-05 | 2.79E-09 | 1.18E-05 | 0 | 0 | 0 | 0.000543 | 0.022792 |
| c__Chloroflexia; | 1.41E-05 | 1.46E-09 | 8.54E-06 | 0 | 0 | 0 | 0.001689 | 0.035471 |
| c__Fibrobacteria; | 0 | 0 | 0 | 1.01E-05 | 1.22E-09 | 1.01E-05 | 0.002781 | 0.038932 |

**Supplementary Table 6** MetaStat of microbes with significant difference between the MA and AH groups.

| Genus level |  |  |  |  |  |  |  |  |
| --- | --- | --- | --- | --- | --- | --- | --- | --- |
| Taxa | mean(MA) | variance(MA) | standard error(MA) | mean(AH) | variance(AH) | standard error(AH) | p value | q value |
| g__Cellvibrio; | 0 | 0 | 0 | 0.0004891 | 2.87E-06 | 0.000489079 | 0.000999 | 0.010348 |
| g__Azospira; | 0 | 0 | 0 | 0.0004454 | 2.34E-06 | 0.000441727 | 0.000999 | 0.010348 |
| g__Helicobacter; | 3.63E-05 | 3.71E-09 | 1.36E-05 | 0.0009866 | 1.85E-06 | 0.000393084 | 0.000999 | 0.010348 |
| g__Alistipes; | 3.33E-05 | 2.15E-09 | 1.04E-05 | 0.0013782 | 2.24E-06 | 0.000431714 | 0.000999 | 0.010348 |
| g__Parabacteroides; | 4.64E-05 | 2.53E-09 | 1.12E-05 | 0.0007261 | 8.60E-07 | 0.000267642 | 0.000999 | 0.010348 |
| g__Parasutterella; | 1.82E-05 | 7.66E-10 | 6.19E-06 | 0.0005244 | 6.01E-07 | 0.000223745 | 0.000999 | 0.010348 |
| g__Cohnella; | 0 | 0 | 0 | 0.0001597 | 3.06E-07 | 0.000159665 | 0.000999 | 0.010348 |
| g__Oscillibacter; | 1.11E-05 | 2.77E-10 | 3.72E-06 | 0.0004672 | 2.97E-07 | 0.000157359 | 0.000999 | 0.010348 |
| g__Faecalibaculum; | 2.22E-05 | 8.52E-10 | 6.53E-06 | 0.000595 | 4.42E-07 | 0.000191919 | 0.000999 | 0.010348 |
| g__Paracoccus; | 0.000164371 | 1.06E-07 | 7.27E-05 | 1.68E-06 | 3.39E-11 | 1.68E-06 | 0.000999 | 0.010348 |
| g__Proteiniphilum; | 8.07E-06 | 1.88E-10 | 3.07E-06 | 0.0002538 | 2.37E-07 | 0.000140663 | 0.001998 | 0.016809 |
| g__Tyzzerella; | 3.03E-06 | 9.74E-11 | 2.21E-06 | 0.000279 | 1.49E-07 | 0.000111599 | 0.000999 | 0.010348 |
| g__Ruminiclostridium; | 6.05E-06 | 2.61E-10 | 3.61E-06 | 0.0002403 | 9.48E-08 | 8.89E-05 | 0.000999 | 0.010348 |
| g__Intestinimonas; | 9.08E-06 | 1.92E-10 | 3.10E-06 | 0.0003126 | 1.16E-07 | 9.84E-05 | 0.000999 | 0.010348 |
